# Supplementary material for: Establishment of a Cell Suspension Culture of Eysenhardtia platycarpa: Phytochemical Screening of Extracts and Evaluation of Antifungal Activity
Source: Plants (Basel). 2021 Feb 23;10(2):414. doi: 10.3390/plants10020414 (PMC7927013; doi:10.3390/plants10020414)
Supplement: Supplementary file 1 [file plants-10-00414-s001.pdf]

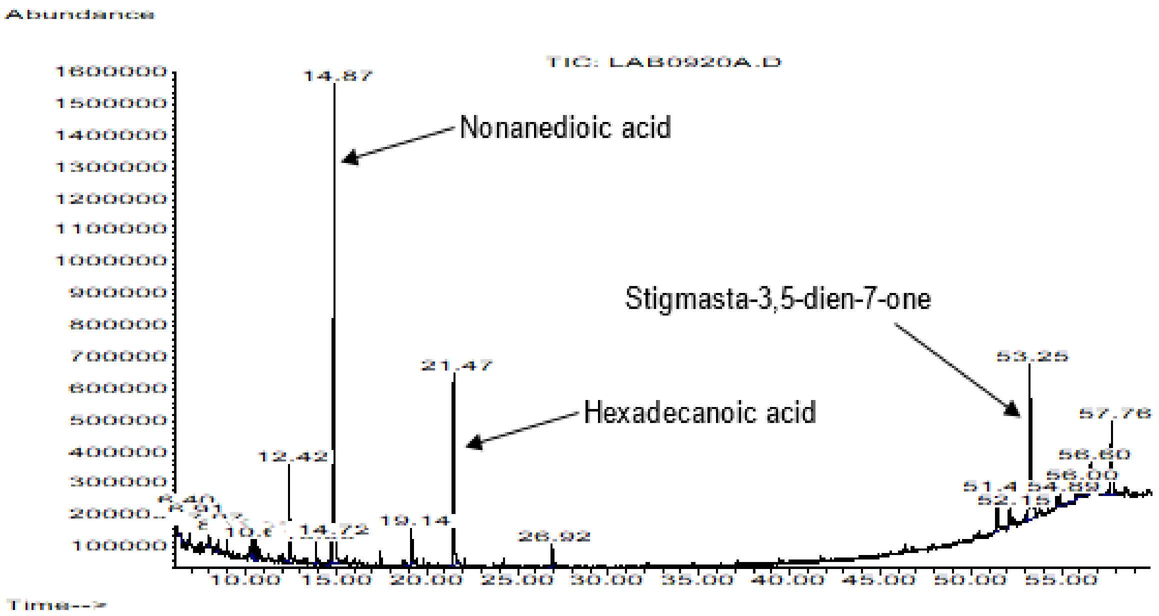

Figure S1. GC-MS chromatogram of the fatty hexane extract of sapwood.

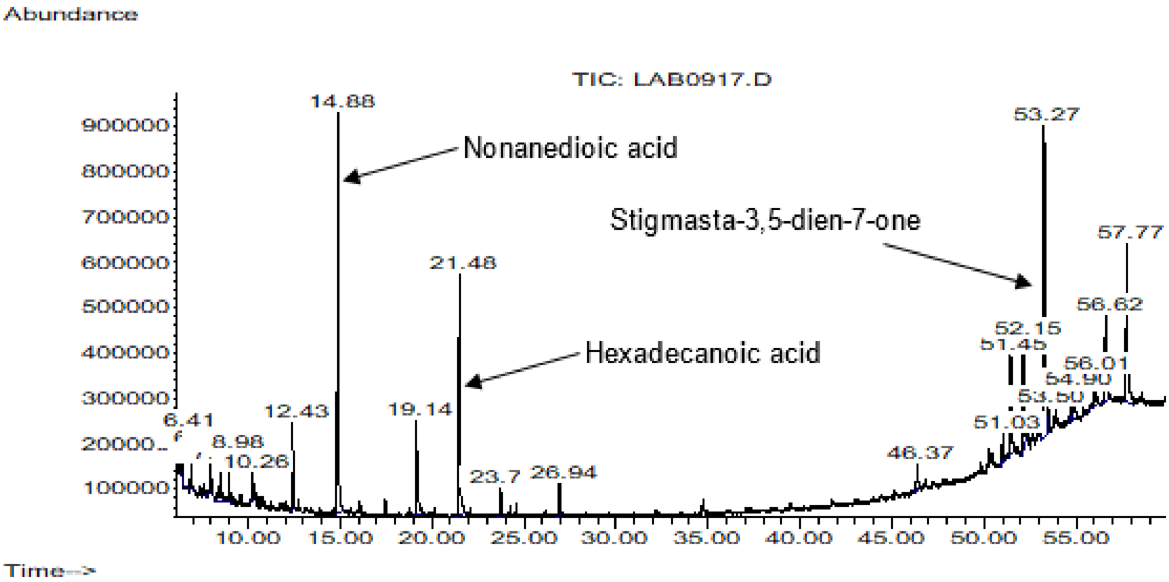

Figure S2. GC-MS chromatogram of the fatty hexane extract of heartwood.

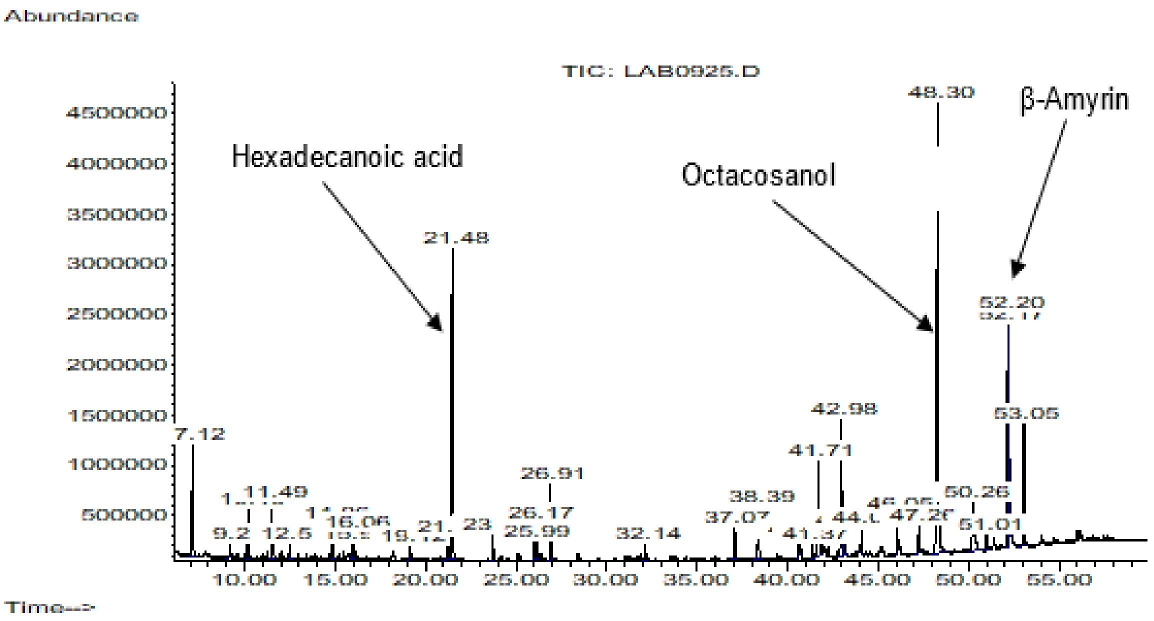

Figure S3. GC-MS chromatogram of the fatty hexane extract of leaf.

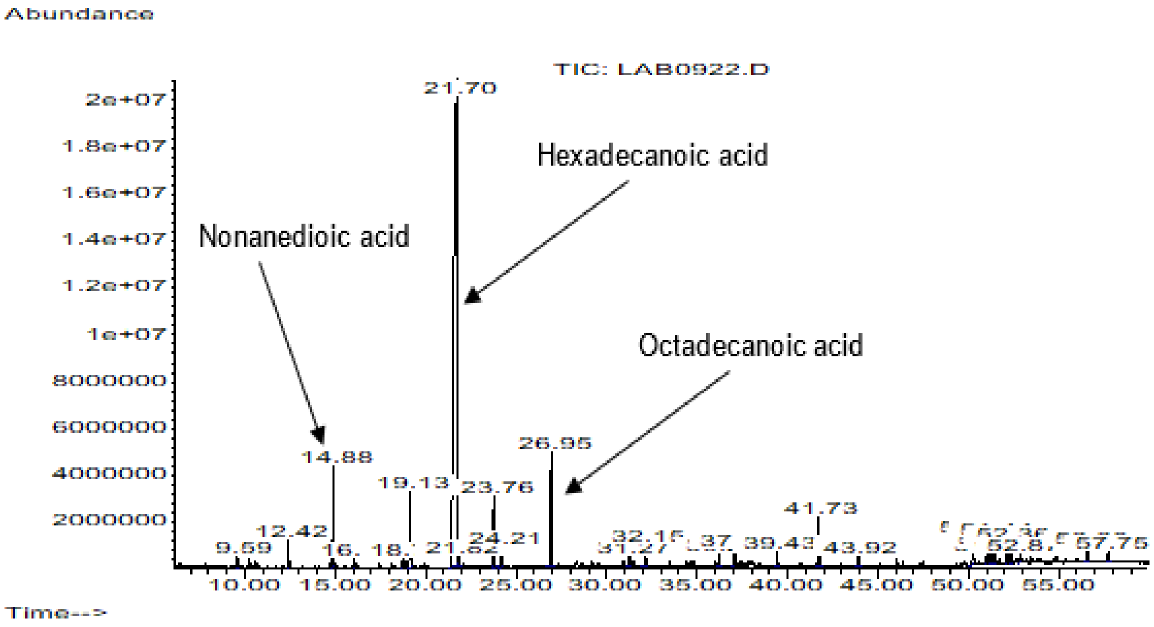

Figure S4. GC-MS chromatogram of the fatty hexane extract of cell suspension cultures.

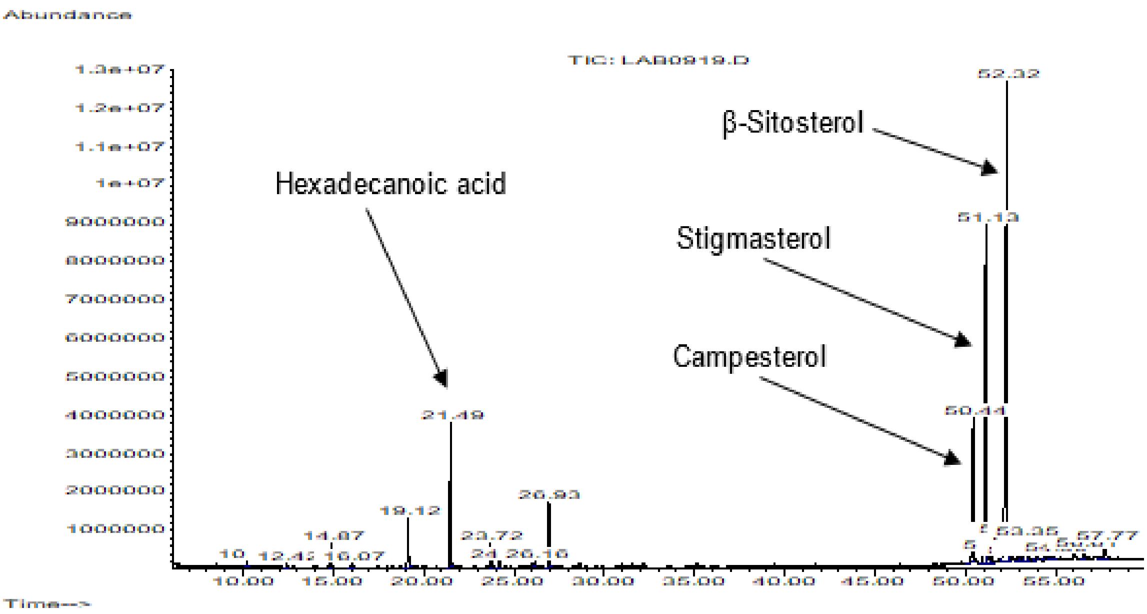

Figure S5. GC-MS chromatogram of the defatted hexane extract of sapwood.

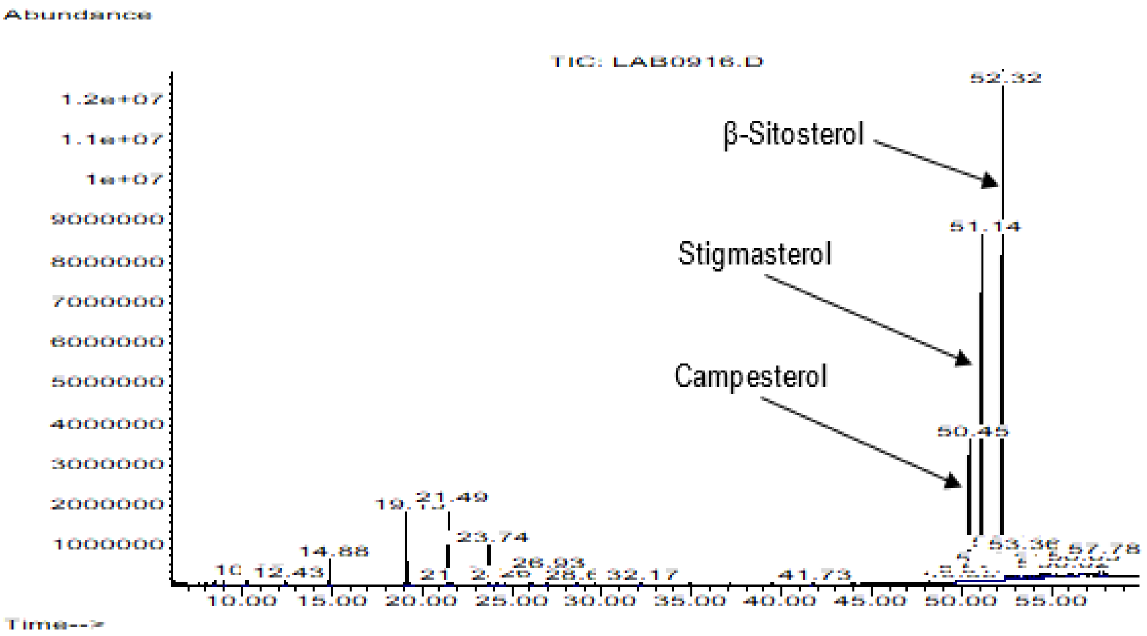

Figure S6. GC-MS chromatogram of the defatted hexane extract of heartwood.

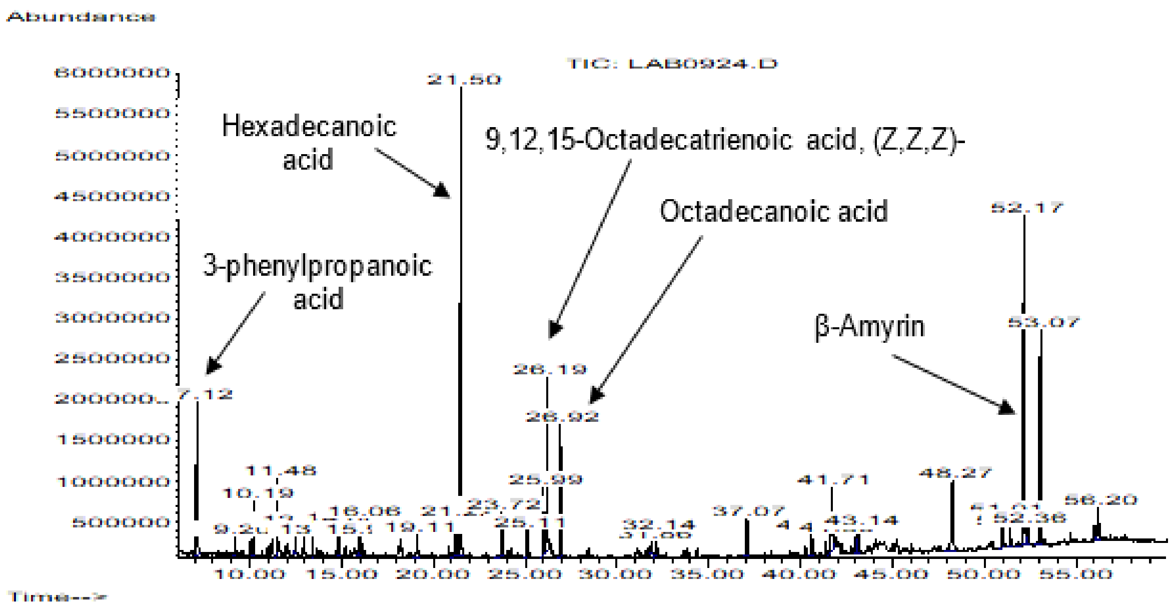

Figure S7. GC-MS chromatogram of the defatted hexane extract of leaf.

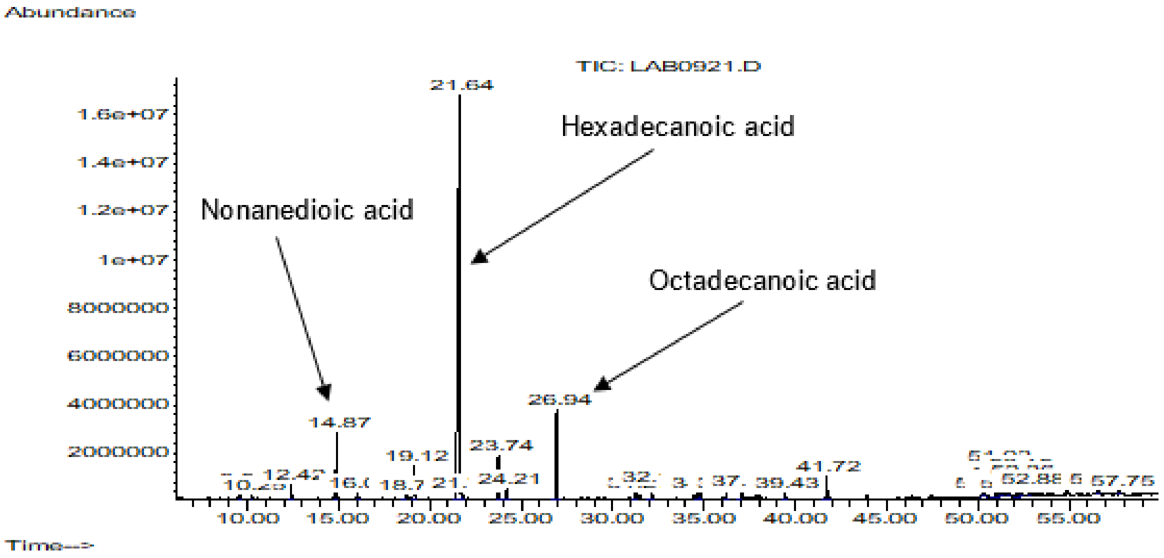

Figure S8. GC-MS chromatogram of the defatted hexane extract of cell suspension culture.

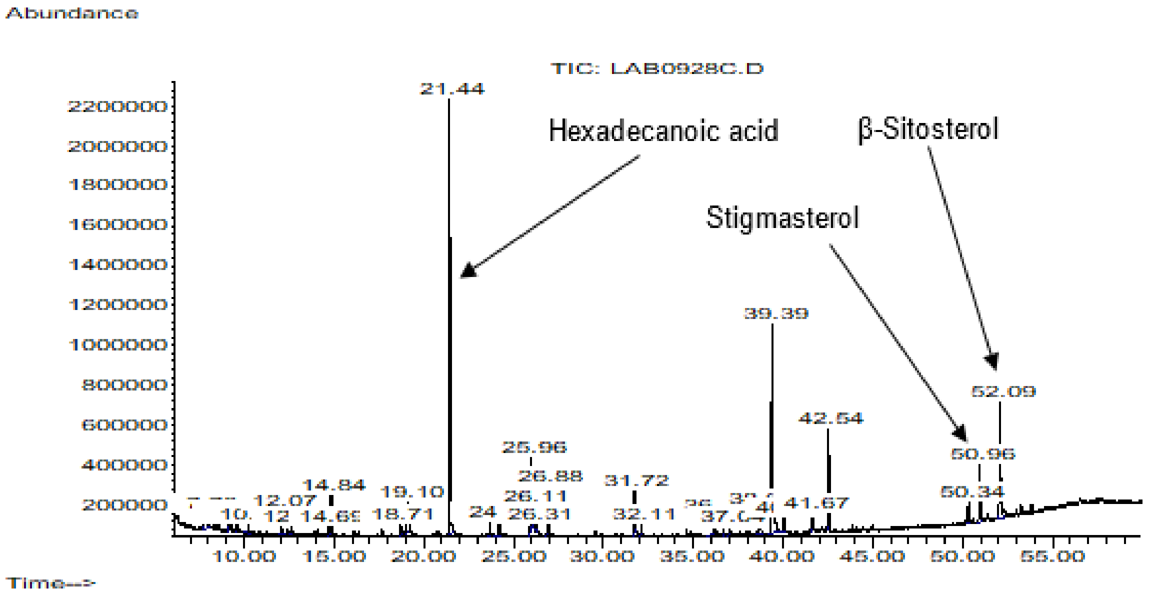

Figure S9. GC-MS chromatogram of the dichloromethane sapwood extract.

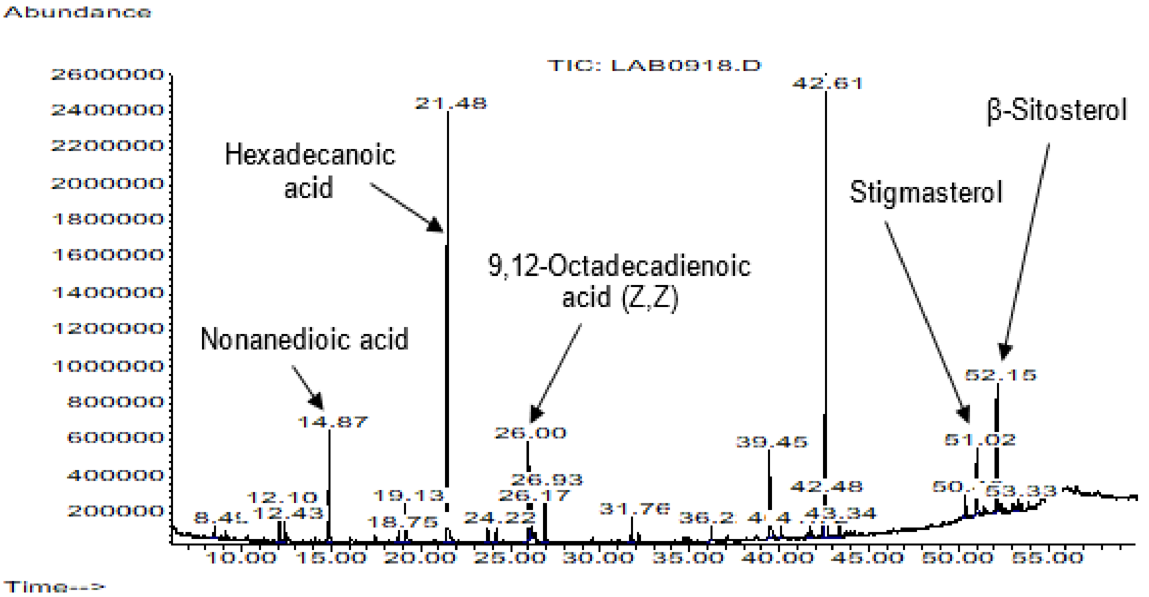

Figure S10. GC-MS chromatogram of the dichloromethane heartwood extract.

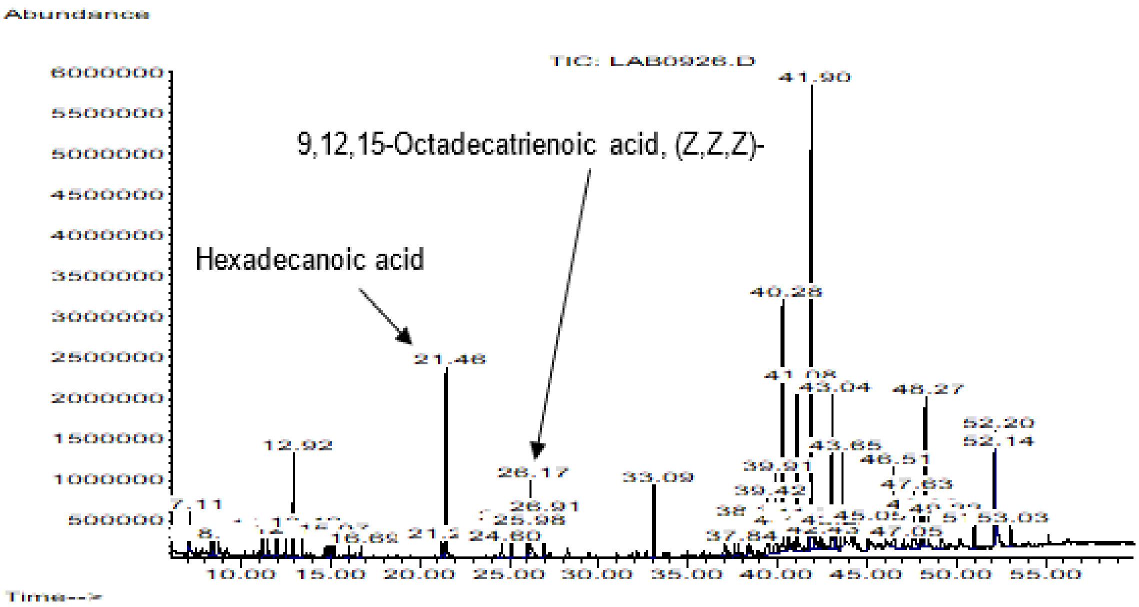

Figure S11. GC-MS chromatogram of the dichloromethane leaf extract.

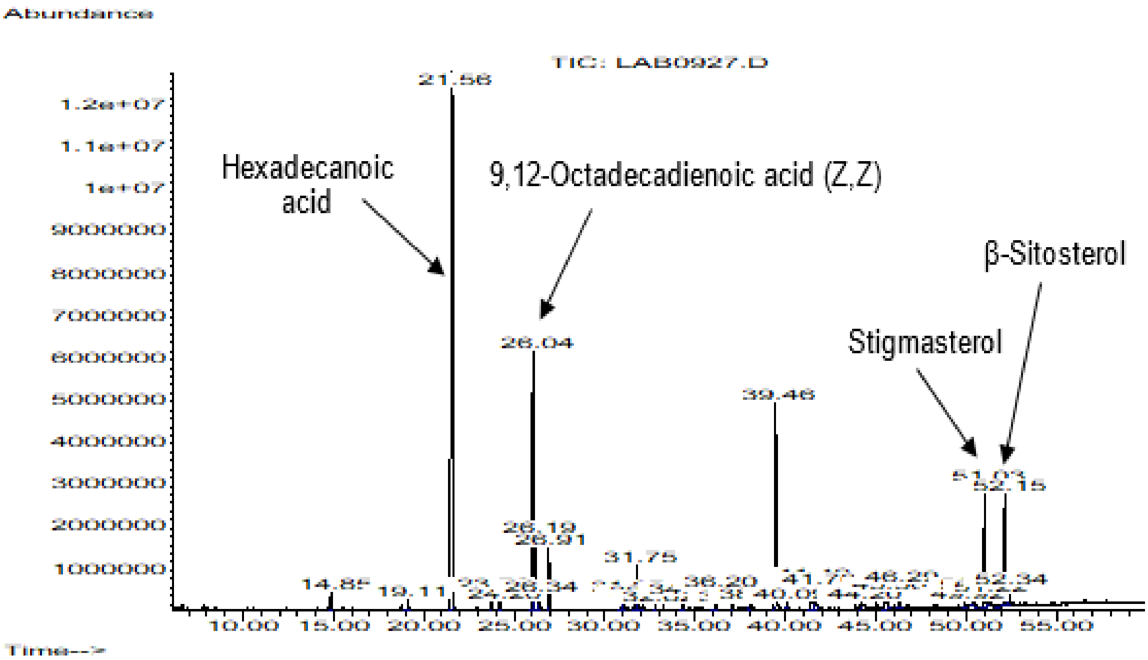

Figure S12. GC-MS chromatogram of the dichloromethane cell suspension culture extract.

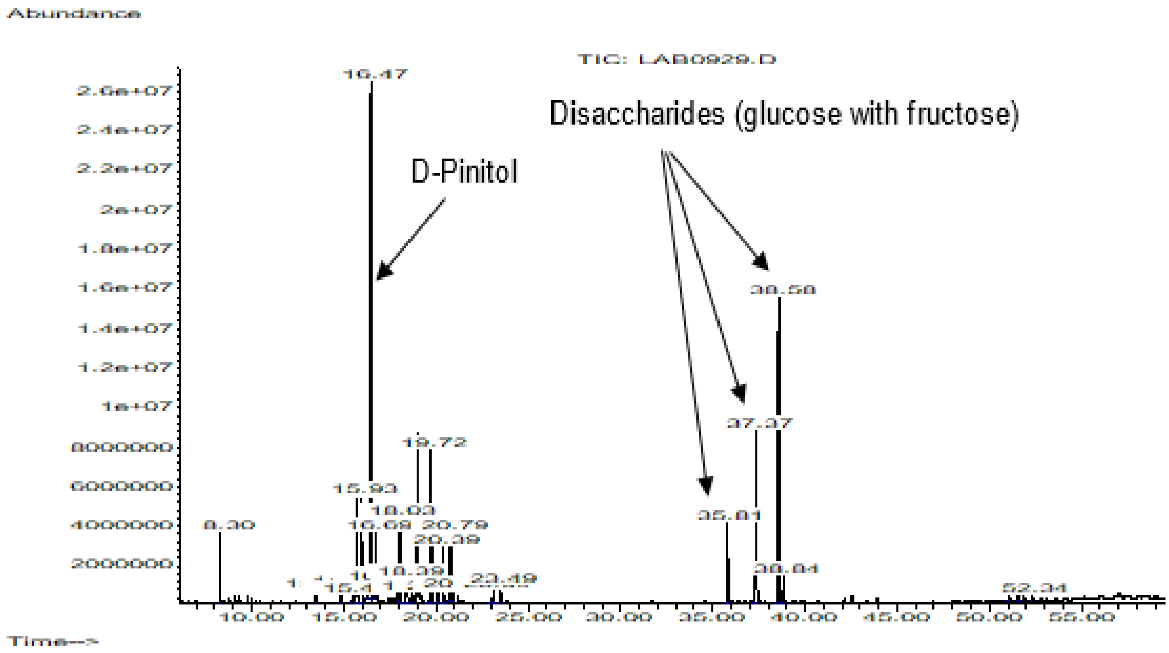

Figure S13. GC-MS chromatogram of the methanolic sapwood extract.

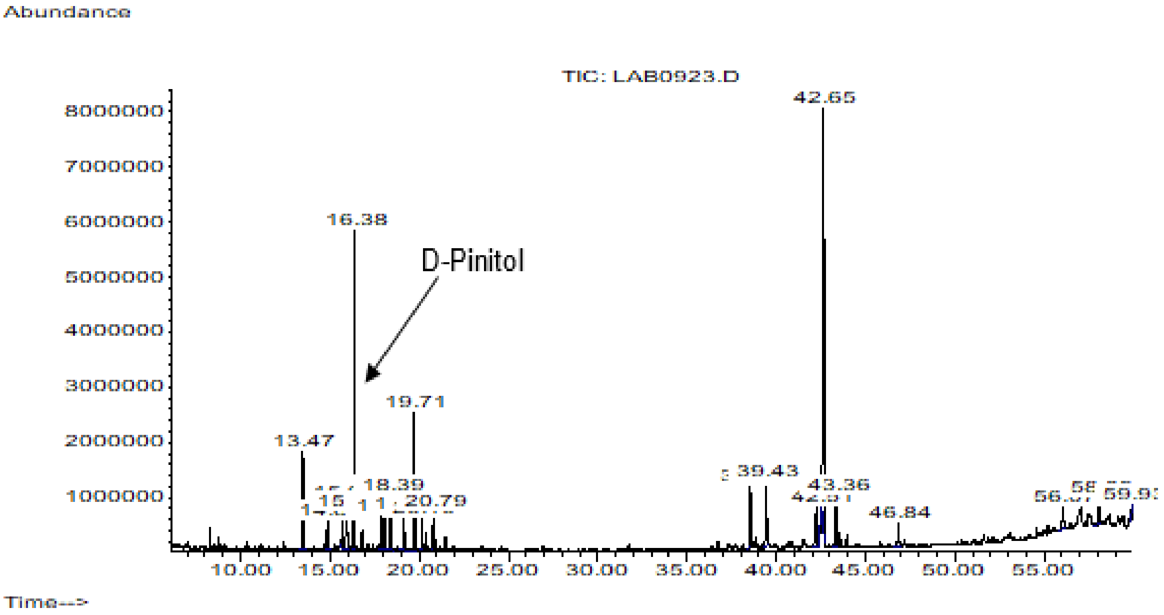

Figure S14. GC-MS chromatogram of the methanolic heartwood extract.

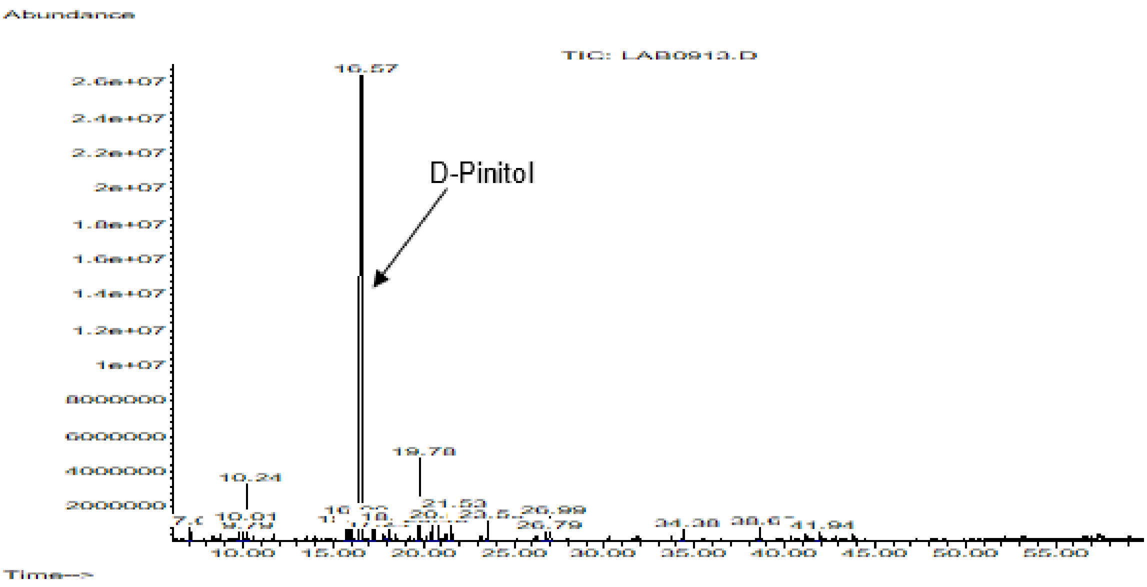

Figure S15. GC-MS chromatogram of the methanolic leaf extract.

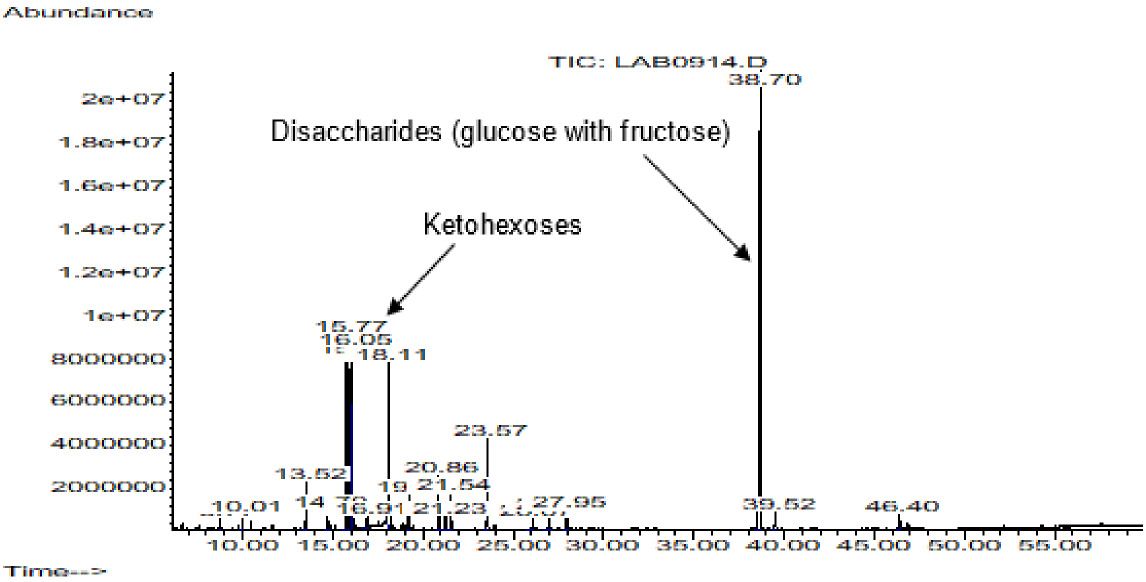

Figure S16. GC-MS chromatogram of the methanolic cell suspension cultures extract.

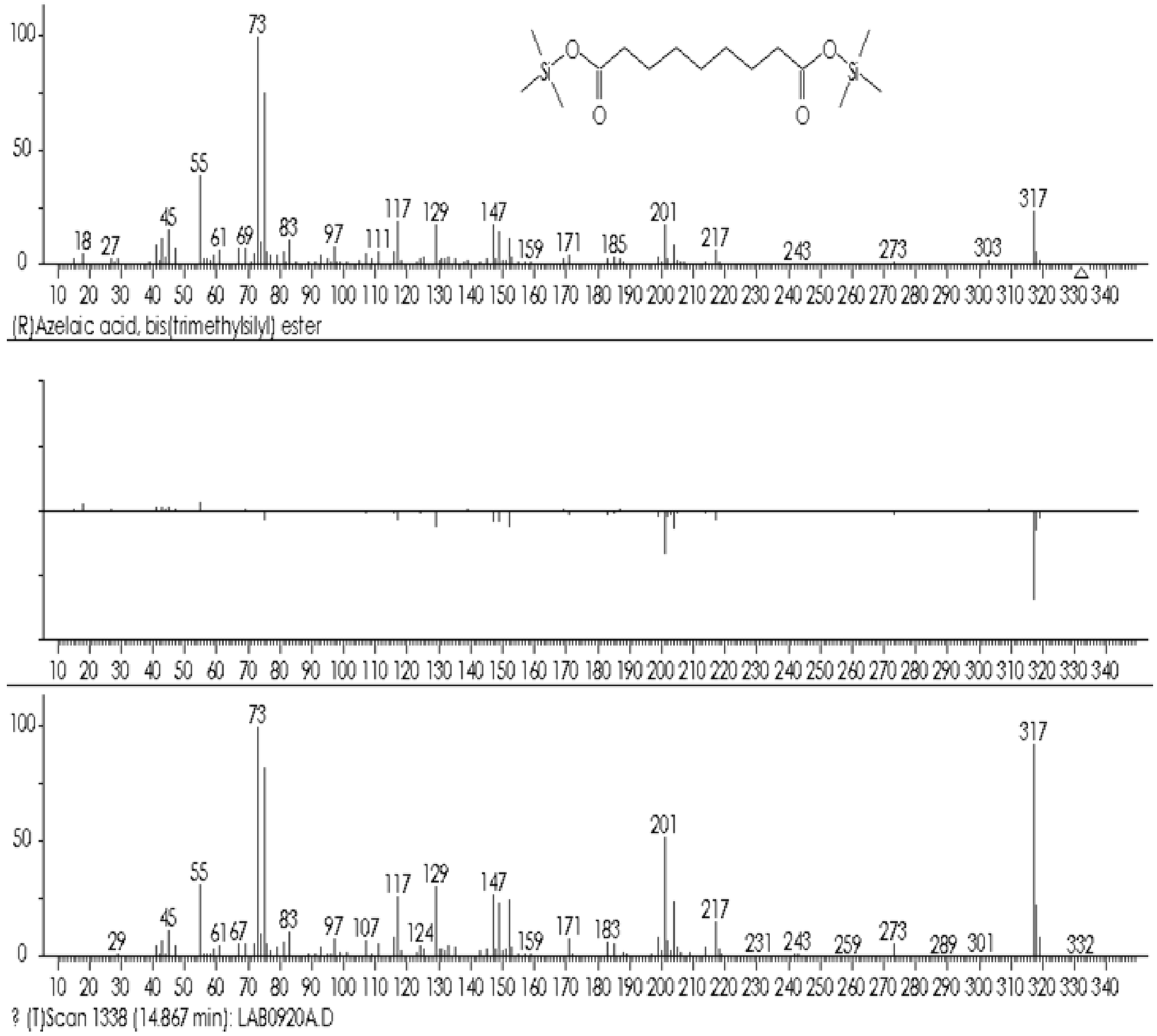

Figure S17. Mass spectrum and structure of nonanedioic acid (azelaic acid).

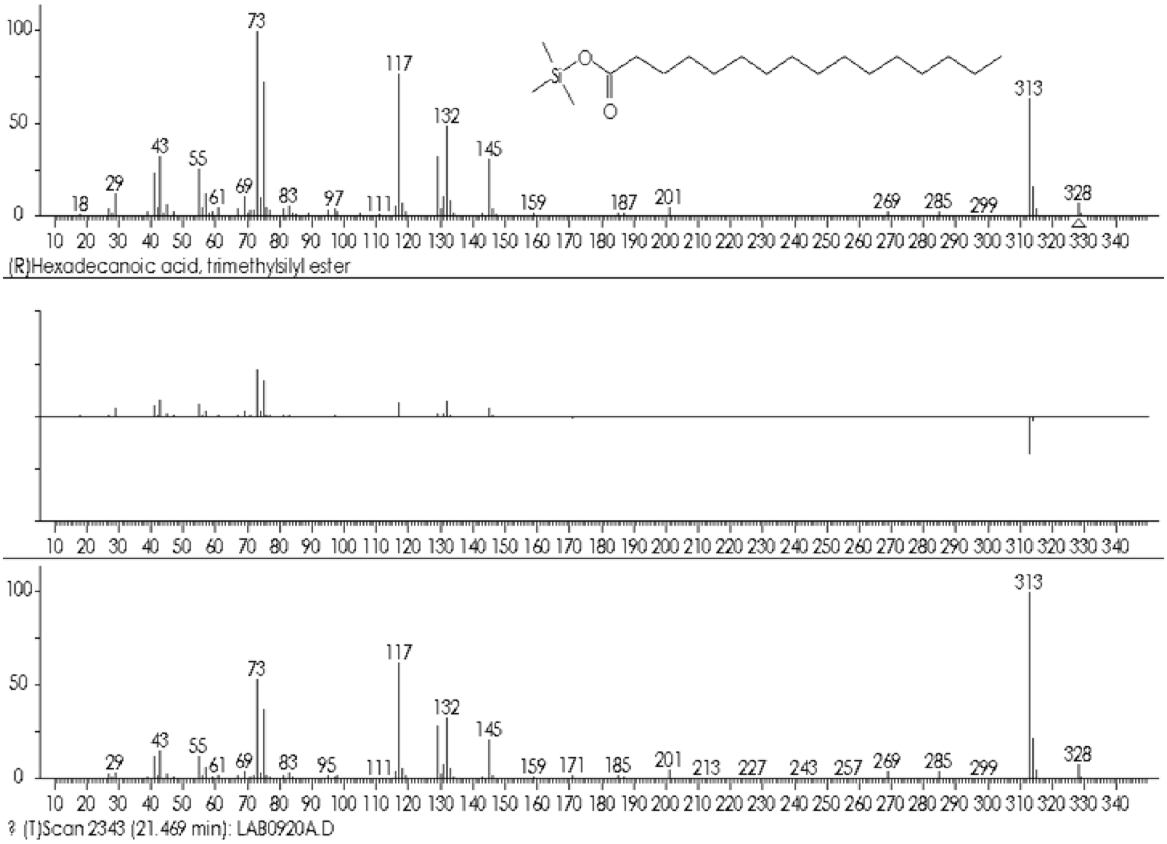

Figure S18. Mass spectrum and structure of hexadecanoic acid.

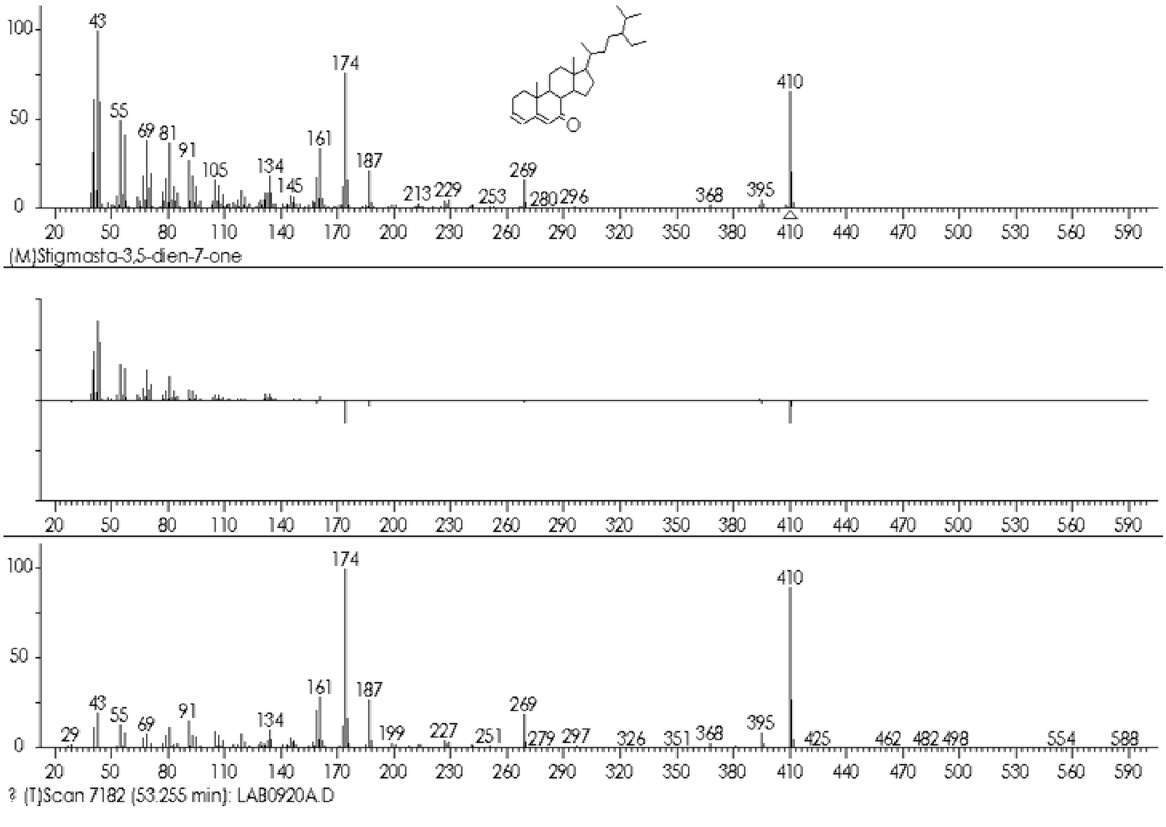

Figure S19. Mass spectrum and structure of stigmasta-3,5-dien-7-one.

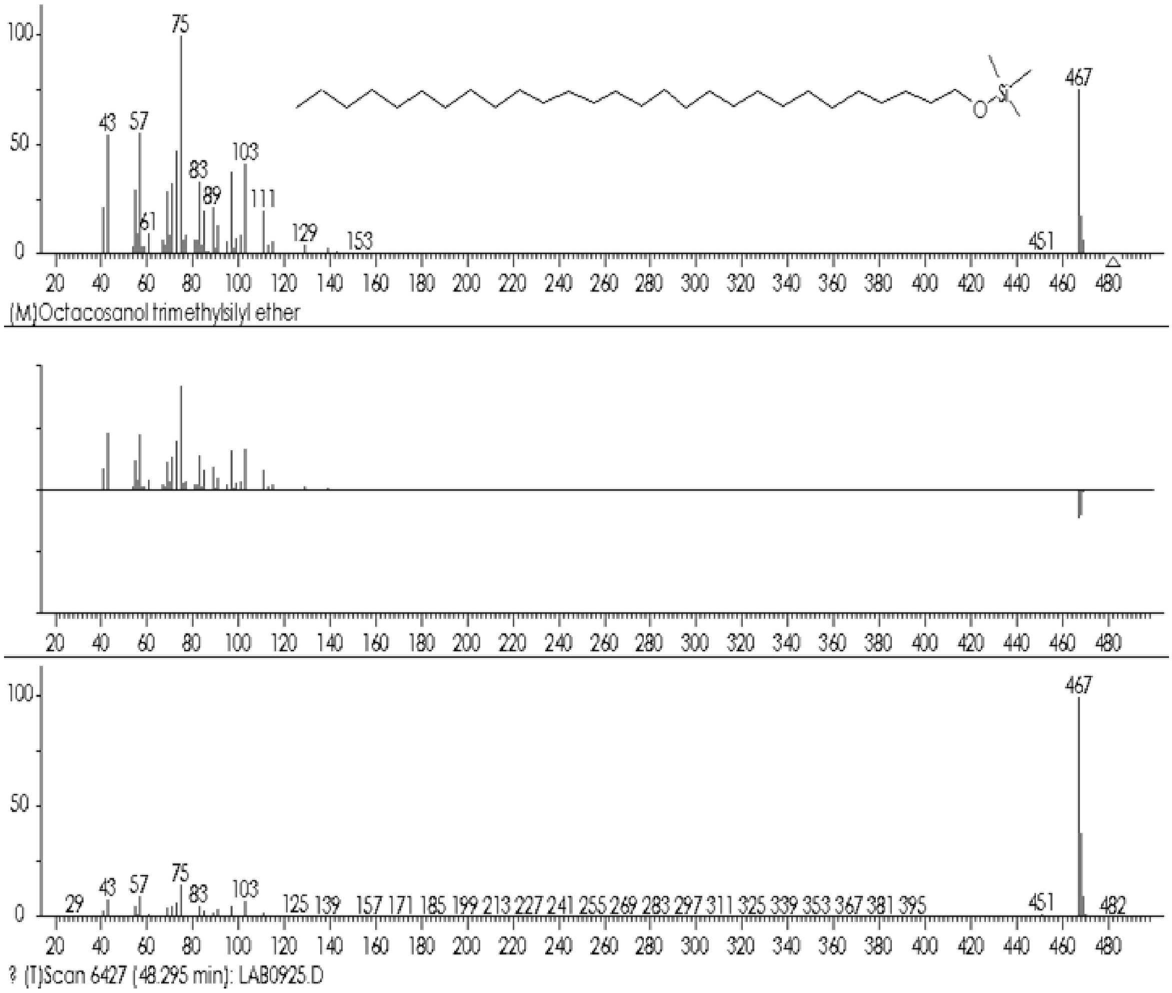

Figure S20. Mass spectrum and structure of octacosanol.

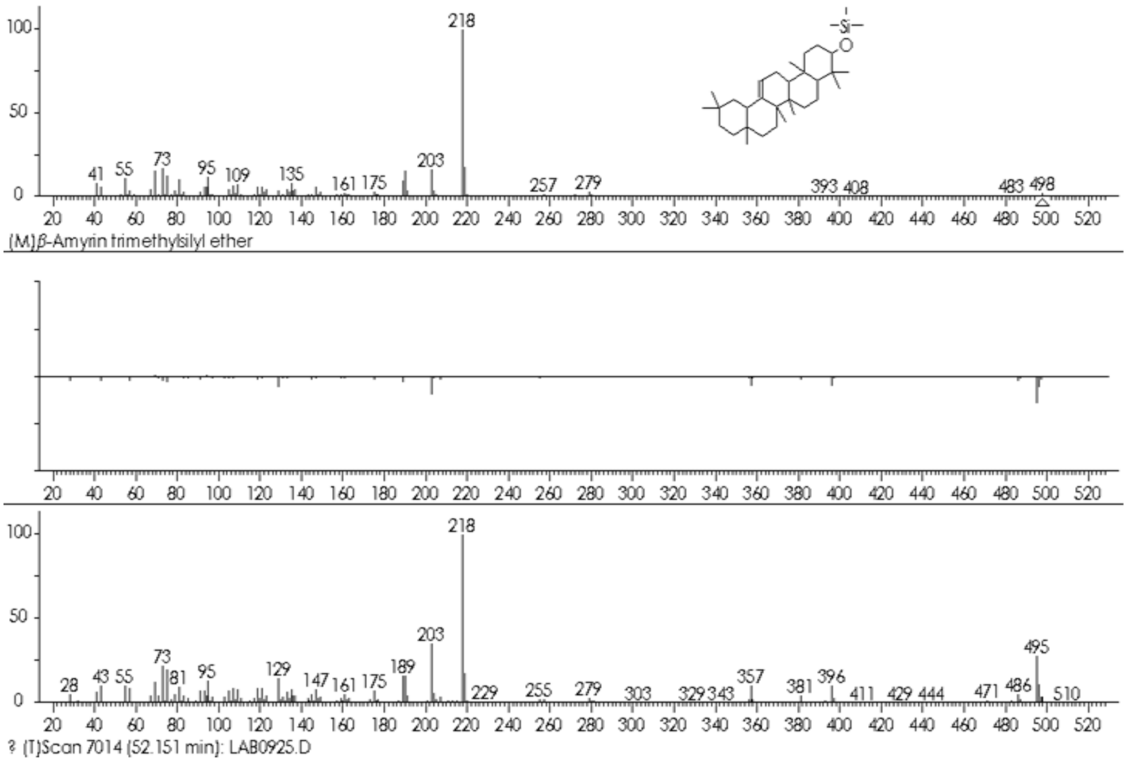

Figure S21. Mass spectrum and structure of *β*-amyrin.

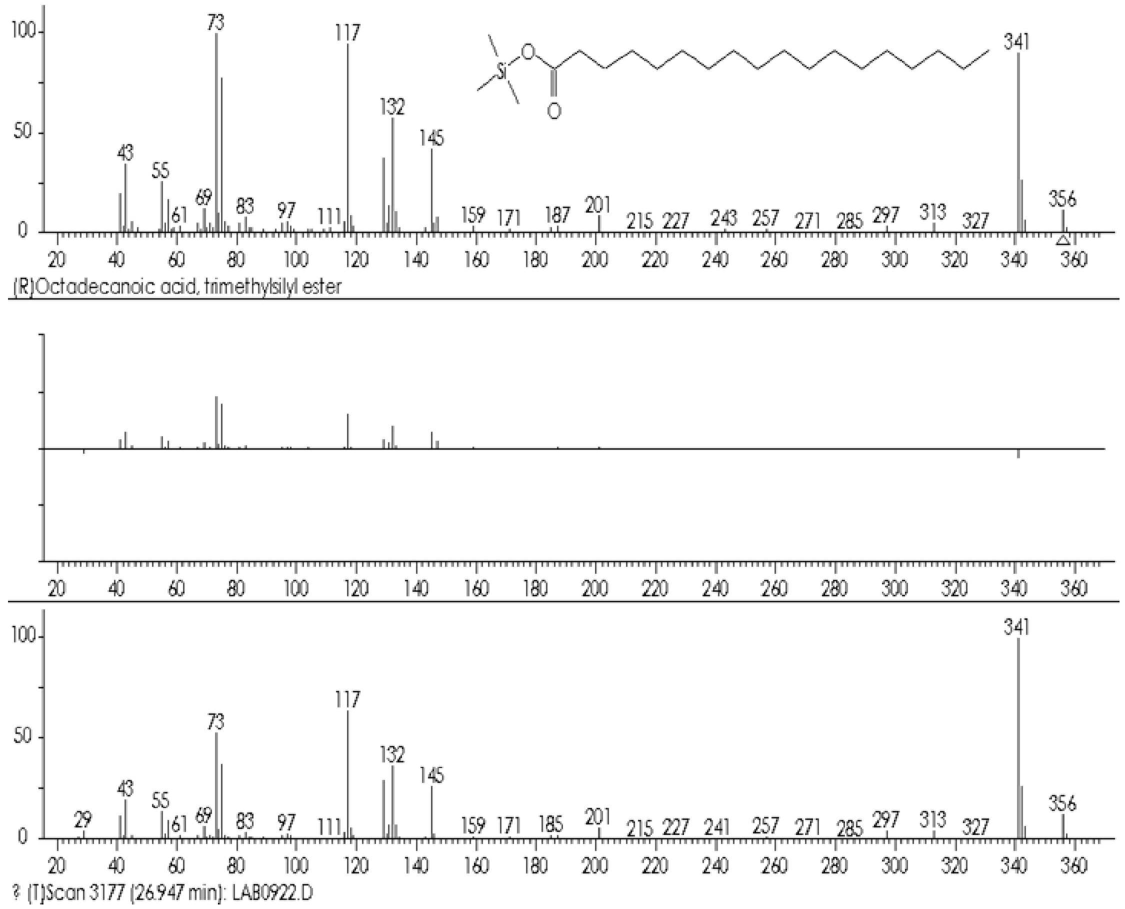

Figure S22. Mass spectrum and structure of octadecanoic acid.

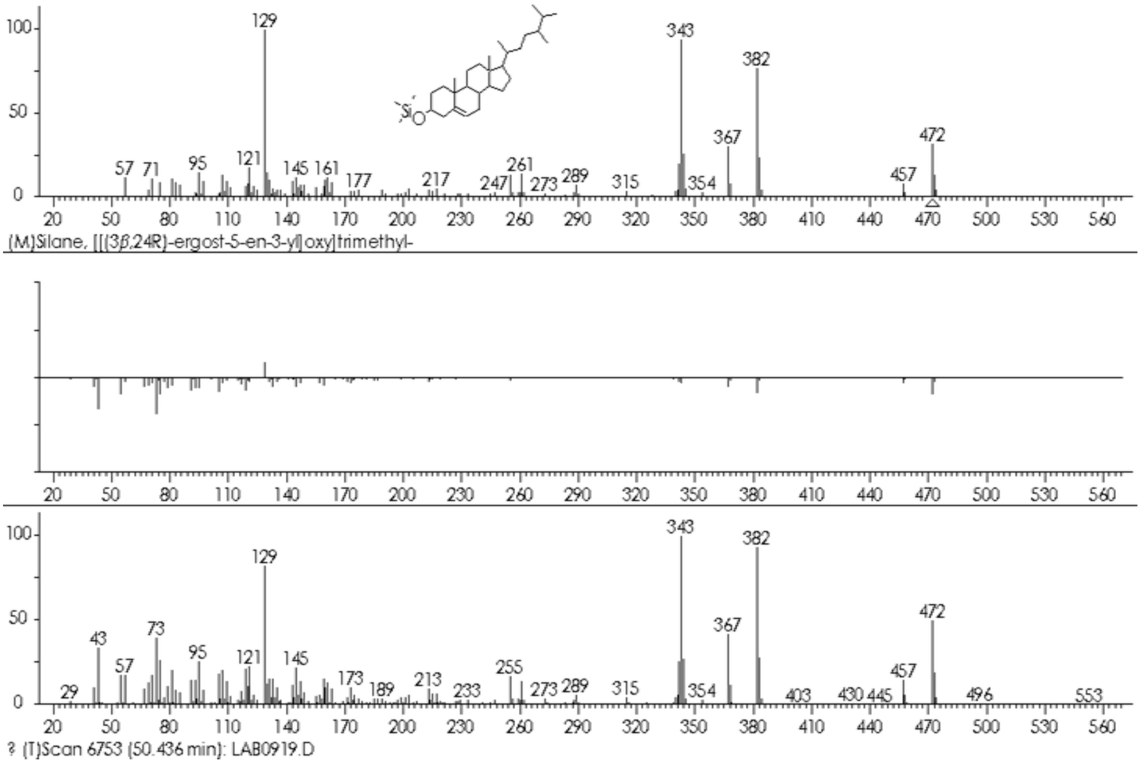

Figure S23. Mass spectrum and structure of campesterol.

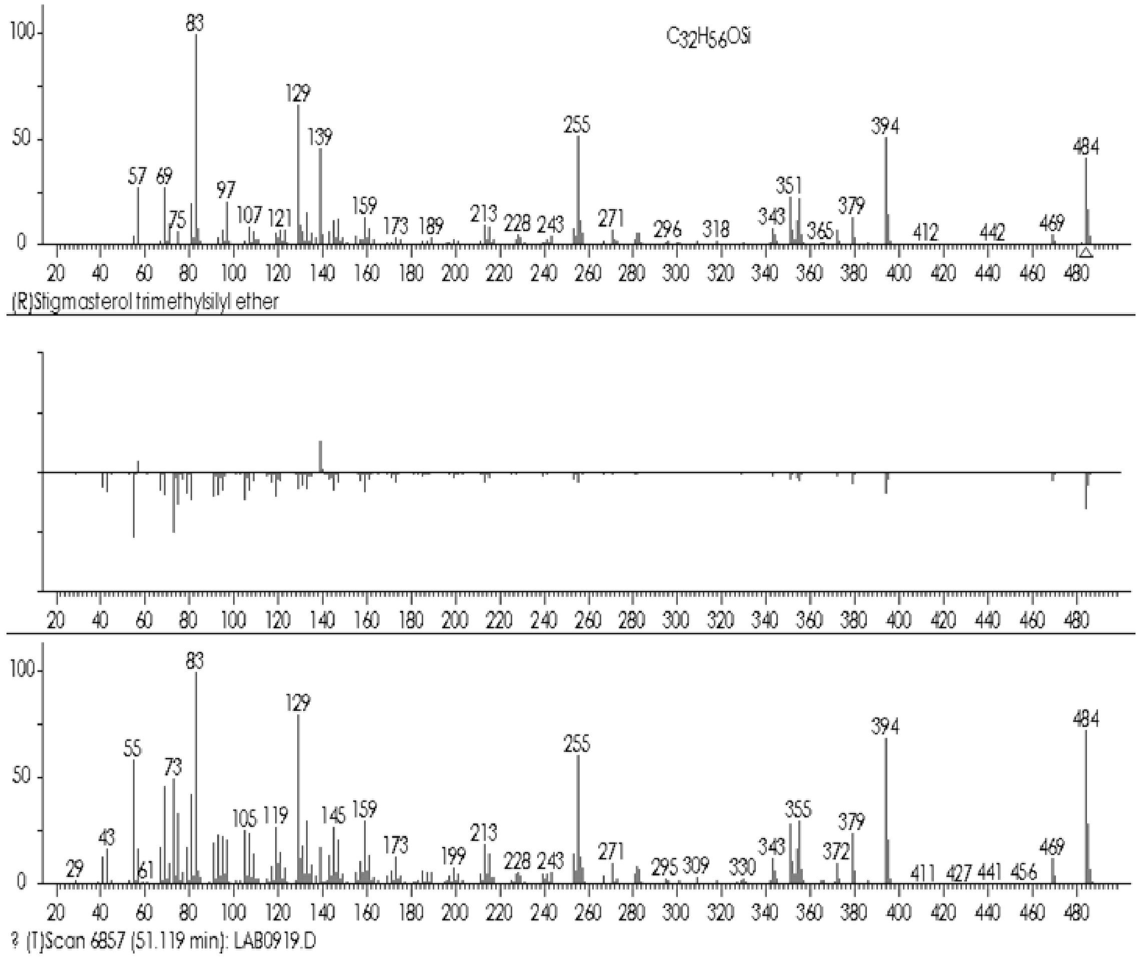

Figure S24. Mass spectrum and structure of stigmasterol.

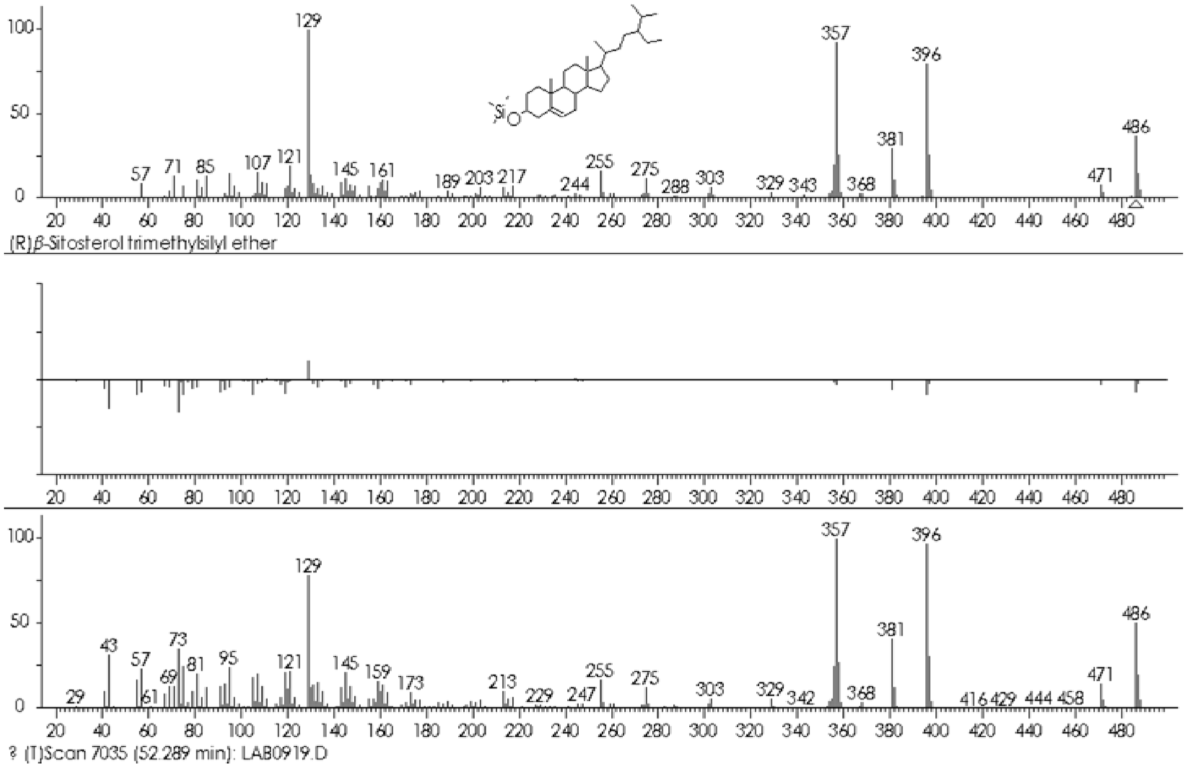

Figure S25. Mass spectrum and structure of *β*-sitosterol.

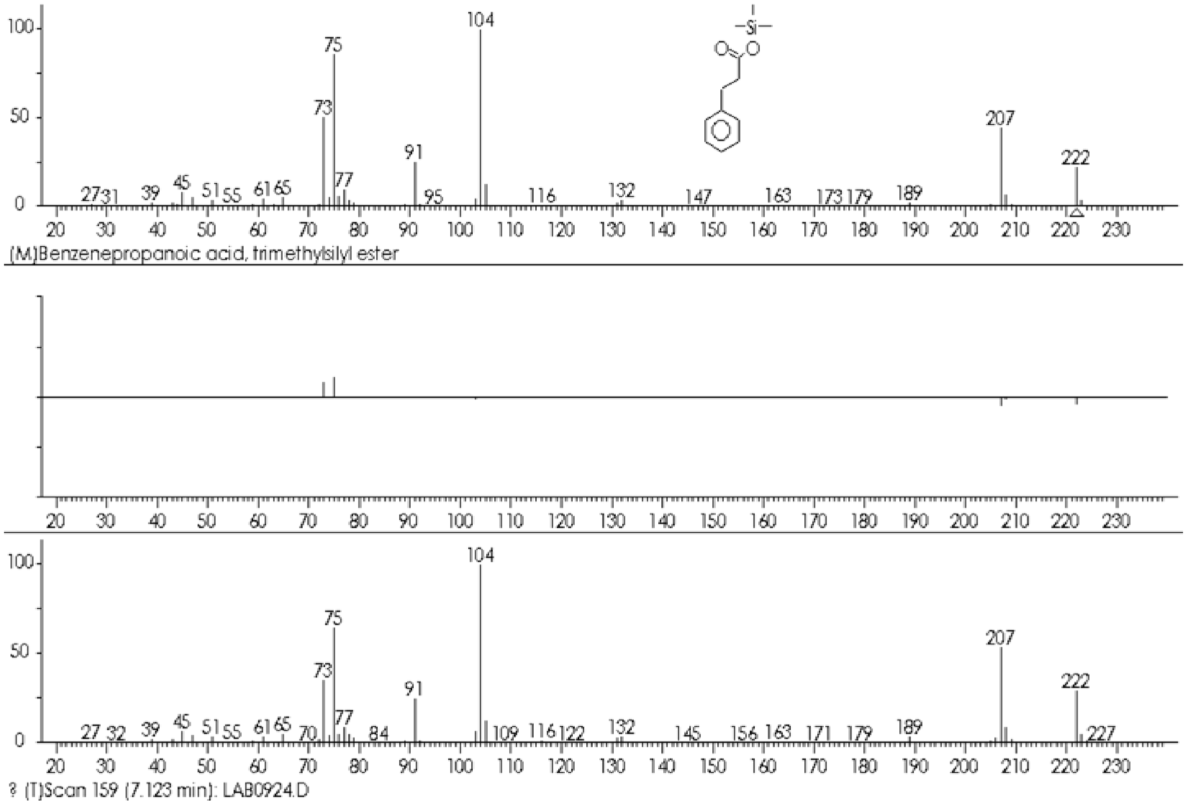

Figure S26. Mass spectrum and structure of 3-phenylpropanoic acid (Benzenepropanoic acid).

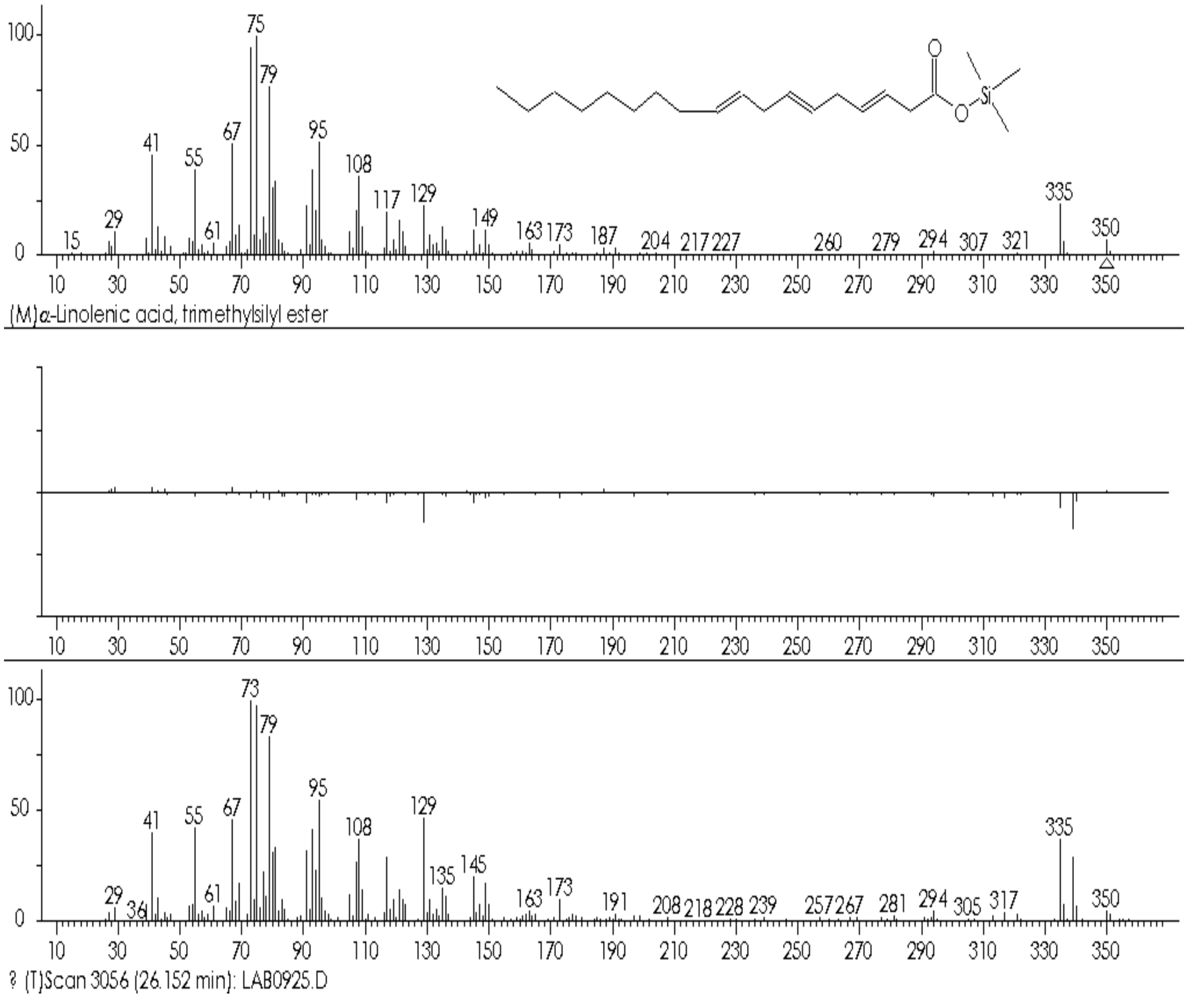

Figure S27. Mass spectrum and structure of 9,12,15-octadecatrienoic acid, (Z,Z,Z)-(α-Linolenic acid).

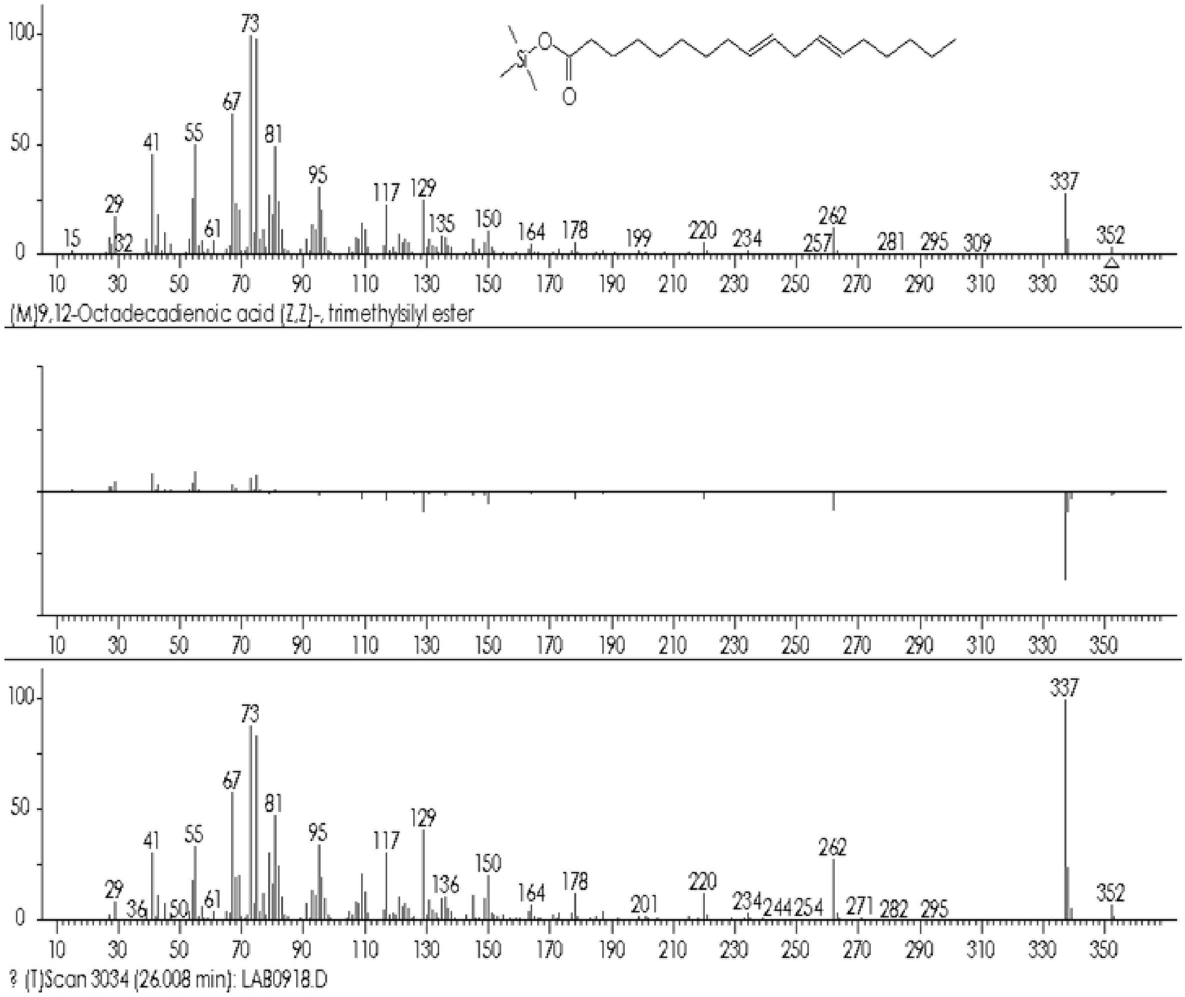

Figure S28. Mass spectrum and structure of 9,12-octadecadienoic acid (Z,Z).

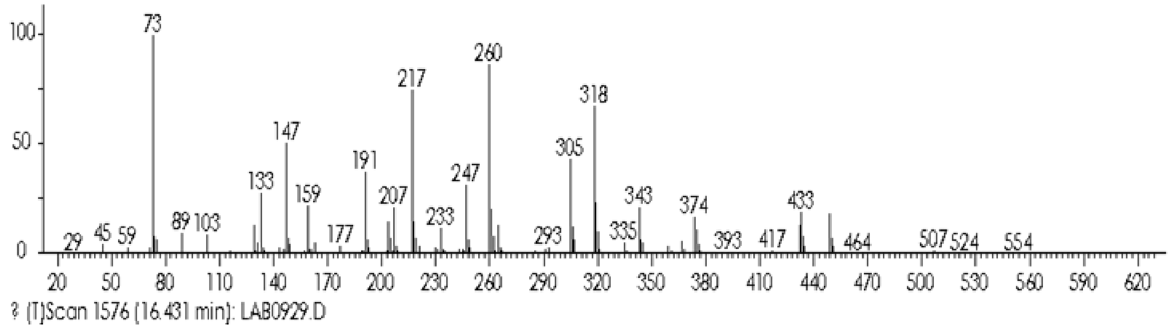

Figure S29. Mass spectrum of D-pinitol, compared with [51].
